# HBM Package Interconnection Pseudo All-Channel Signal Integrity Simulation and Implementation Method of the Synchronous Current Load Research

**DOI:** 10.3390/mi16080896

**Published:** 2025-07-31

**Authors:** Wen-Xue Tang, Cong-Jian Mai, Li-Yan Zhou, Ying Sun, Xin-Ran Zhao, Shu-Li Liu, Gang Wang, Da-Wei Wang, Cheng-Qian Wang

**Affiliations:** 1 China Electronics Technology Group Corporation 58th Research Institute, Wuxi 214122, China; robbie_tang999@aliyun.com (W.-X.T.); njuzly@163.com (L.-Y.Z.); h2436333461@163.com (Y.S.); officebomb@163.com (X.-R.Z.); liushuli_1990@126.com (S.-L.L.); wanggang_cetc@126.com (G.W.); 2 Innovation Center for Electronic Design Automation Technology, School of Electronics and Information, Hangzhou Dianzi University, Hangzhou 310005, China; 232040273@hdu.edu.cn

**Keywords:** HBM, signal integrity, SSO, SSI, Pseudo full-channel simulation, synchronized current loads, CCCS

## Abstract

This paper proposes a pseudo full-channel signal integrity (SI) simulation method tailored for high-bandwidth memory (HBM) interconnects. In this approach, real interconnect models are applied to selected portions of the channel, while the remaining sections are replaced with synchronized current loads that emulate the electrical behavior of actual signal transmission. This technique enables accurate modeling of the HBM interface under full-channel parallel data transfer conditions. In addition to the simulation methodology itself, this study focuses on three specific implementation schemes for the synchronized current loads and explores their practical applications. Comparative analysis demonstrates the necessity and effectiveness of using synchronized current loads as substitutes for real transmission loads, offering a viable and efficient solution for SI analysis in HBM interconnect systems.

## 1. Introduction

High-bandwidth memory (HBM) is a type of dynamic random-access memory (DRAM) module that adopts a multi-layer stacked structure. It has advantages such as high capacity and high bandwidth and has become the most promising memory solution in artificial intelligence (AI) computing chips. In contrast, traditional DRAM chips, such as Double Data Rate (DDR) DRAM and Graphics Double Data Rate (GDDR) DRAM, are limited in terms of bandwidth and capacity density, and can no longer meet the memory performance demands of large-scale data processing in AI. The latest generation of DRAM standard, DDR5, released in July 2020, provides a maximum capacity of 8 GB (64 Gb), a data rate of 6.4 Gbps per pin, and a 64-bit bus width [1]. By comparison, the HBM3 standard released in January 2022 supports up to 16-layer stacking, with a single module offering up to 64 GB total capacity, which is eight times that of DDR5. The data rate is also 6.4 Gbps per pin, but the bus width reaches 1024 bits, and its parallel transmission bandwidth can be up to 16 times that of DDR5 [2]. It can be seen that HBM significantly outperforms traditional DDR in key performance indicators such as capacity and bandwidth, making it an ideal memory solution for high-performance computing systems.

The significant improvement in HBM transmission bandwidth mainly relies on the increase in parallel transmission bus width. The HBM2 and HBM3 series of high-bandwidth memory, which are widely used in AI computing chips, both feature an effective parallel transmission bus width of 1024 bits. The HBM4 standard, released in April 2025, further enhances transmission capability by doubling the effective parallel bus width of a single die to 2048 bits [3]. Unlike traditional DDR modules, which use PCB-level assembly and interconnect technologies, HBM, due to its high-density and thousand-scale interconnect requirements, is typically integrated with AI computing chips in a 2.5D advanced packaging structure. Typical packaging methods include Chip-on-Wafer-on-Substrate (CoWoS) with an interposer [4,5,6,7], and Embedded Multi-die Interconnect Bridge (EMIB), which uses embedded silicon bridges for multi-chip interconnection [8,9,10].

In the packaging and integration design of HBM and AI computing chips, one of the key objectives is to achieve ultra-high interconnect bandwidth between the two. This relies on detailed layout and routing design, as well as multiple rounds of verification and optimization. Common verification methods include comprehensive signal integrity (SI) and power integrity (PI) simulation analyses of the packaging interconnects [11,12]. For parallel transmission interfaces such as traditional DDR or HBM, which use source-synchronous clocking, signal integrity simulations typically adopt parallel synchronous transmission simulation methods, including two modes: synchronous switching output (SSO) and synchronous switching input (SSI), corresponding to write and read operations from the processor to the memory, respectively. However, performing SSO and SSI simulations for the interconnect between HBM and AI computing chips presents significant challenges. Whether using electromagnetic field solvers (EM Solvers) to extract scattering parameter models (S-Parameters) of the interconnect structure, or time-domain simulations based on Simulation Program with Integrated Circuit Emphasis (SPICE), both approaches require extremely high computational resources and long simulation times, posing serious challenges to design efficiency and development cost. To address these challenges, the industry is exploring various technical directions. For example, Cadence, a leading global EDA vendor, has introduced the Clarity EM Solver tool optimized for efficient computation in 2.5D high-density interconnects, which significantly improves the solving efficiency of interconnect models for heterogeneous integration packaging, reducing simulation time by a certain factor or proportion [13,14], and is particularly well-suited for extracting S-Parameters models of HBM-to-AI chips interconnects. The method proposed in this paper specifically targets the interconnect structures between HBM and AI computing chips, and aims to address the SSO and SSI scenarios by proposing a time-domain SPICE simulation method that balances simulation accuracy and efficiency, thereby seeking breakthroughs at the simulation methodology level.

SSO and SSI simulations not only reflect the impact of interconnect channel factors such as reflection and loss on signal integrity, but more importantly, they accurately characterize the synchronous switching noise (SSN) caused by crosstalk between a large number of parallel signals during toggling and transmission, as well as by parasitic effects in the I/O power delivery network (PDN) [15,16]. Moreover, the power noise induced by SSN can further lead to signal jitter, known as power supply noise induced jitter (PSIJ) [17], which is directly manifested in the eye diagram during simulation. Therefore, conducting SSO and SSI simulations for the interconnect structures between HBM and AI computing chips must comprehensively consider the effects of signal crosstalk and the I/O interface PDN on signal integrity. Essentially, SSO and SSI simulations are a type of SI-PI co-simulation method, which integrates I/O models, signal delivery network (SDN) models, and PDN models.

**Figure 1:**
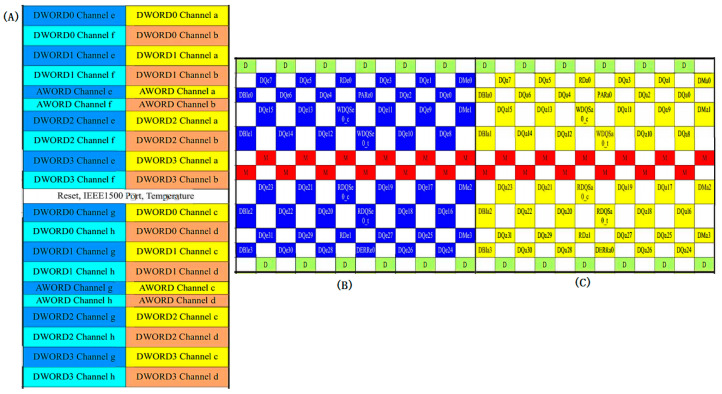
(**A**) HBM2/HBM2e ball-out footprint (not to scale), (**B**) Channel e Dword0 ball-out footprint, and (**C**) Channel a Dword0 ball-out footprint [18].

In the HBM2/HBM2e standards, all 1024 valid data signals (DQ) of the HBM interface are divided into eight independent physical channels, each equipped with its own address and control unit. Within each channel, the 128-bit DQ are further divided into four Dwords, with each Dword consisting of 32 DQ along with the corresponding auxiliary signals, forming the smallest synchronous transmission unit in HBM data transfer, totaling 48 interconnect signals. Each Dword is equipped with independent read/write data strobe signals (RDQS/WDQS) for source-synchronous clock transmission and reception control, as shown in Figure 1. In the simulation method proposed in this paper, two adjacent Dwords are grouped together to form a unit with coupling between signals, which is used as the target for interconnect modeling and simulation, while the remaining units maintain the same interconnect design structure. During SSO and SSI simulations of the HBM interface, to balance simulation accuracy and resource consumption, only selected unit groups are subjected to full signal transmission simulation. Moreover, to accurately reflect the impact of power SSN and PSIJ—generated by simultaneous I/O switching activity on the PDN—on signal integrity when the entire 1024-bit signal and its synchronous clock are operating concurrently, those unit groups not modeled with actual interconnects must be loaded with functional workloads to emulate the full-channel operation scenario. This ensures that the proposed method significantly improves simulation efficiency while maintaining accuracy.

## 2. Pseudo Full-Channel SSO/SSI Simulation Method

**Figure 2:**
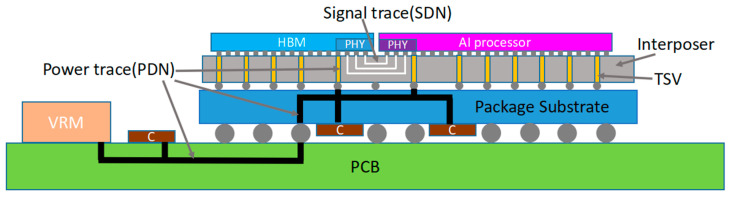
CoWoS-S packaging integration structure of HBM and AI computing chips.

The 2.5D packaging integration structure of the HBM and AI computing chips studied in this paper is shown in Figure 2. The packaging assembly consists of a package substrate and a silicon-based interposer with through-silicon vias (TSV). All signal interconnections between the HBM and AI chips are implemented in the metal layers of the interposer. The PDN and decoupling network of the HBM interface physical layer (PHY) I/O are delivered through four hierarchical levels: the system board (PCB), the package substrate, the interposer, and inside the PHY, ultimately connecting to the I/O drivers and receivers. The packaging design employs power and ground planes that effectively connect all vertical and horizontal power delivery paths, reducing PDN impedance and suppressing SSN, thereby ensuring stable power supply under high parallel transmission conditions and minimizing the impact of power noise on signal integrity.

**Figure 3:**
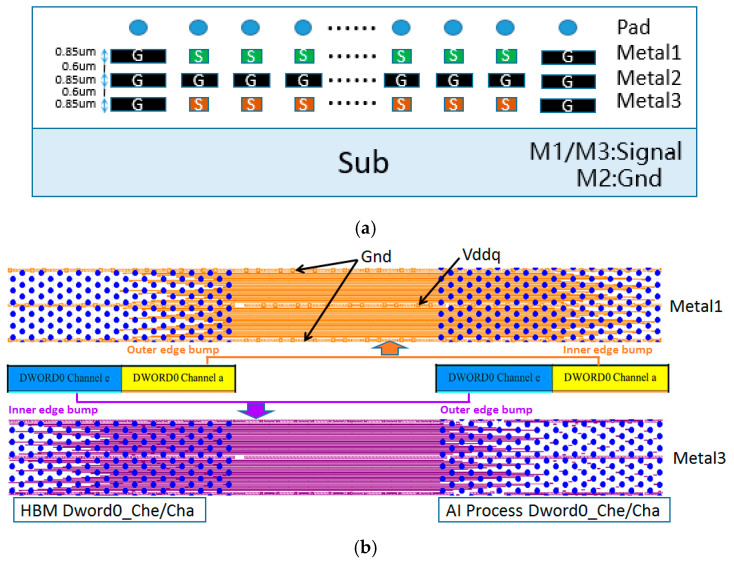
(**a**) Cross-sectional view of the metal line structure in the interconnect unit group and (**b**) layout wiring for metal interconnect structure of the first and third layer in the unit group.

The metal interconnections of the HBM interface signals are located within the interposer layer. In this design, two Dwords located in the same row along the vertical direction at the die edge of the HBM interface PHY (as shown in Figure 1: Channel e Dword0 and Channel a Dword0) are defined as the smallest interconnect unit group with signal coupling, while the remaining 15 pairs of adjacent Dword unit groups in the same row maintain an identical metal interconnect structure. As shown in Figure 3a, the interconnect structure utilizes four metal layers on the interposer; excluding the pad layer, the remaining three metal layers are used for signal routing. The M1 layer is used to route signals from Channel a Dword0, connecting bumps located on the outer edge of the HBM chips to bumps on the inner edge of the AI computing chips, while the M3 layer routes signals from Channel e Dword0, connecting bumps from the inner edge of the HBM chip to the outer edge of the AI chips. The M2 layer serves as a common GND layer, providing return current paths and signal isolation. As illustrated in Figure 3b, the signal routing direction is perpendicular to the chips edge, following a uniform line width and spacing rule of 2 μm/4 μm (Line/Space), while the GND layer follows a 3 μm/3 μm routing rule. Additionally, at the boundary between unit groups, a GND network with a spacing of twice the bump pitch is implemented to serve as vertical shielding between unit groups, thereby effectively suppressing inter-group crosstalk and minimizing its impact on signal integrity to a negligible level.

**Figure 4:**
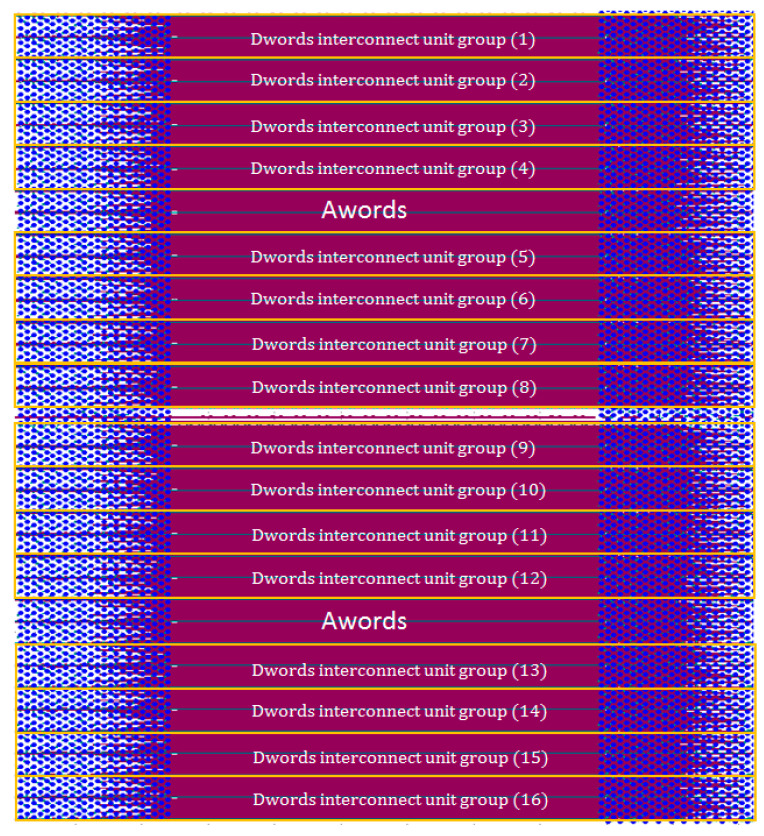
All interconnects divided into 16 repeatable Dwords interconnect unit groups (excluding Aword).

The unit group interconnect design shown in Figure 3 is based on a thorough consideration of the HBM layout structure. It reflects the signal coupling relationship between two adjacent Dwords in the same row, while structural isolation is used to minimize the impact of signal crosstalk between different Dword unit groups in the vertical direction. Based on presented construction, the crosstalk between vertically adjacent Dwords unit is at least below −35 dB, and the value makes the coupling between data signals uninfluential. Each unit group contains two Dwords, comprising a total of 64-bit DQ data signals, and including auxiliary signals such as address, control, and clock signals; the total number of interconnect signals reaches 96. These are distributed across the upper and lower metal layers (Metal1 and Metal3), with 48 interconnect lines per layer. Given that all 16 interconnect unit groups share an identical structure, when extracting interconnect models using an EM solver, the 96 interconnect lines of a single unit group can be modeled and extracted as one unified model, and this model can be reused to represent the interconnect structures of the other unit groups if needed, thereby significantly reducing modeling workload and improving simulation efficiency. As shown in Figure 4, this reusable Dword interconnect unit group serves as the fundamental building block of the entire simulation method.

**Figure 5:**
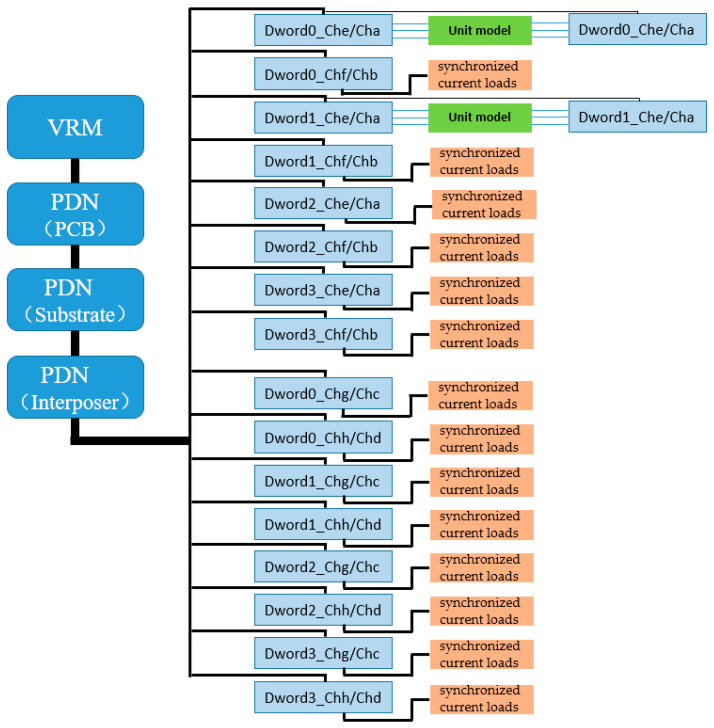
Pseudo full-channel synchronous signal transmission simulation interconnect topology for HBM (2+14).

According to the multipath power delivery principle of the PDN, all vertical and horizontal power supply paths are interconnected in the package through power and ground planes, which can effectively reduce PDN impedance. Therefore, in simulation, a complete HBM interface power PDN model (full 16 channels) must be included to ensure that the simulation circuit accurately reflects the power load conditions when all Dword units in the HBM interface operate simultaneously, thereby realistically representing the SSN induced during the simultaneous toggling of the 1024-bit data signals and their auxiliary signals, and further characterizing its impact on PSIJ. Based on the 16 interconnect unit groups defined between the HBM and AI computing chips on the interposer, all of which have identical interconnect structures, and considering the above PDN simulation requirements and constraints, this paper proposes a pseudo full-channel synchronous signal transmission simulation circuit interconnect topology, as shown in Figure 5. In this topology, two interconnect unit groups (Channel e Dword0/Dword1 and Channel a Dword0/Dword1) are selected to construct real interconnect units between the HBM and AI chips, with real HBM I/O models connected at both the driver and receiver ends. A PRBS11 pseudo-random bit sequence is applied at the driver end, and a full power delivery path is established by connecting the PCB, package substrate, and interposer PDN models (including decoupling capacitors) in series at the I/O power terminals, along with the chip-internal power network model (Chip Power Model, CPM) placed near the I/O. For the remaining unit groups, instead of using interconnect line models and I/O drivers, synchronous current load models are used to replace the actual current loads generated during real Dword operation, thereby ensuring that the PDN experiences equivalent current flow as in full-channel parallel operation. In this simulation, a 2+14 configuration is adopted, consisting of 2 real interconnect units and 14 pseudo interconnect units (i.e., synchronous current load models), and the units connected via synchronous current loads are defined as “pseudo interconnect units.”

## 3. Implementation Methods and Simulation Schemes of Synchronous Current Loads

Although the 1024 bits of the HBM interface are divided into eight completely independent channels, during AI large-scale data access, the read and write commands of these eight channels are logically synchronized. Therefore, in SSO and SSI simulations of full-channel signal transmission for the HBM interface, while the logical states of the 1024 bits change randomly, their state transitions must remain synchronous. To simulate the current variations in a Dword during operation using a current load, it is essential to ensure that this current load is fully synchronized with the actual current variations occurring during the real Dword operation; thus, this paper defines it as a “synchronous current load.” Furthermore, the closer the dynamic current waveform of the synchronous current load matches the real I/O switching current, the higher the accuracy of the simulation results. In transient simulation, the toggling of the 64-bit DQ within a single unit group (comprising two Dwords) is driven by applying different pseudo-random binary sequences (PRBS11 in this paper) to each I/O input, generating logic transitions. The simultaneous toggling of these 64 I/O signals causes current surges (boost or drop), which linearly superpose to form the dynamic current flowing on the PDN, ultimately constituting the current variation waveform of the synchronous current load.

In summary, the synchronous current load must satisfy the following two constraints: (1) the dynamic current variation in the load must be synchronized with the toggling of the logic data driving the Dword I/O; (2) the total current load of the unit group must be the linear superposition of the current changes generated by multiple I/O. Based on these constraints, this paper implements the synchronous current load using a Current Controlled Current Source (CCCS) and proposes three different CCCS synchronous current load models, each applied in the pseudo full-channel synchronous signal transmission simulation method presented herein. The following sections will sequentially introduce the design methods of these three CCCS models and their specific applications in simulation.

### 3.1. Proportional Linear (Linear) CCCS Synchronous Current Load and Simulation Configuration Scheme

**Figure 6:**
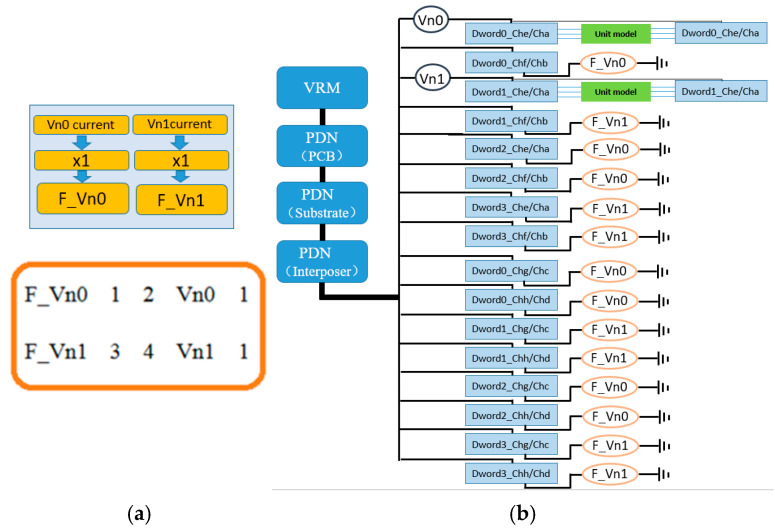
(**a**) Proportional linear CCCS synchronous current load and (**b**) simulation configuration scheme.

**Figure 7:**
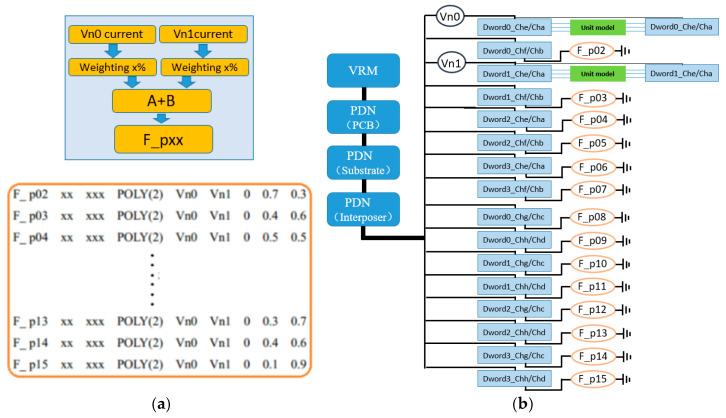
(**a**) Polynomial weighted sum CCCS synchronous current load and (**b**) simulation configuration scheme.

The proportional linear CCCS synchronous current load implementation method proposed in this paper is shown in Figure 6. In the simulation circuit, two 0 V voltage sources (Vn0 and Vn1) are connected in series with the I/O power supply terminals (VDDQ) of the two real interconnect unit groups. During simulation, all DQ and auxiliary signals within these two unit groups are transmitted synchronously in the transient simulation through interconnect models, and the I/O drive currents in each unit group flow losslessly through the two 0 V voltage sources. The currents flowing through these two voltage sources are then used to control two CCCS components (F_Vn0 and F_Vn1) with a 1:1 proportional ratio. These CCCS outputs are subsequently connected to the PDN power supply ports of the remaining 14 pseudo interconnect units that do not include interconnect models, as shown in Figure 7. In this configuration, the pseudo interconnect units do not require I/O driver models, but the CPM for the I/O drivers on the driving side must still be integrated into the power delivery network. Additionally, the simulation netlist must ensure that the currents flowing through Vn0 and Vn1 fully represent the total current entering the I/O driver power ports after decoupling within the die.

In this example, the two controlled current sources F_Vn0 and F_Vn1 are configured with a gain ratio of exactly 1:1 relative to the current flowing through the control sources Vn0 and Vn1. The network connection relationships and parameter implementation syntax of the controlled current sources are as follows:

(1) F_Vn0 1 2 Vn0 1

F_Vn0 is the name of the controlled current source. Nodes 1 and 2 represent the connection points of F_Vn0 within the simulation circuit. Vn0 is the current control source, and the value 1 indicates the gain. Here, the gain is set to 1, meaning that the current output by the controlled source F_Vn0 has the same magnitude as the current flowing through the control source Vn0. Since this example uses a linear source without delay, the above statement indicates that the current of F_Vn0 is fully synchronized with the current through Vn0, both in magnitude and phase. The implementation of F_Vn1 is identical to that of F_Vn0, with its current control source being Vn1.

In the simulation configuration scheme shown in Figure 6b, among the 16 interconnect unit groups, 2 units use real interconnect models, while the remaining 14 pseudo interconnect units are divided such that 7 even-numbered units (Dword0/Dword2) use the F_Vn0 synchronous current load and 7 odd-numbered units (Dword1/Dword3) use the F_Vn1 synchronous current load. This configuration effectively divides the 16 units into two groups, with each group of 8 units sharing a current load of identical magnitude and phase. These eight groups of current loads flow simultaneously through the entire PDN and decoupling network. Each unit has the same current load, which means that, at any given moment of logic transition, there are identical numbers of bits toggling from 0 to 1 and from 1 to 0 across the units. Considering an extreme case: if, at a particular logic transition moment in the unit where Vn0 is located, all 64 bits toggle from 0 to 1, then the other seven units using the F_Vn0 synchronous current load will also effectively experience 64-bit 0-to-1 transitions. This results in a linear superposition of the dynamic current required for 512 bits switching from 0 to 1, flowing through the PDN. In summary, the primary feature of this method is that it increases the likelihood of peak dynamic current occurring in the PDN and decoupling network. As a result, this method is probabilistically suited for simulating the worst-case power noise on the PDN and the maximum PSIJ on signals. However, from the perspective of dynamic current generated by the entire 1024-bit toggling, the randomness of bit transitions is relatively reduced in this approach.

### 3.2. Polynomial Weighted Sum (Poly) CCCS Synchronous Current Load and Simulation Configuration Scheme

The polynomial weighted sum (Poly) CCCS synchronous current load and its simulation configuration scheme are illustrated in Figure 7. The synchronous current loads connected to the 14 pseudo interconnect units are implemented using a polynomial weighted sum approach. This type of CCCS generates the target current signal by applying weighted superposition to the control source currents captured from Vn0 and Vn1. During the weighting process, the phase of the current signals remains unchanged, meaning the controlled source currents remain fully synchronized with those of Vn0 and Vn1—with only the amplitude being scaled. Multiple weighted currents are then linearly summed to form a new composite output current. For example, F_p02 represents a new current source formed by combining 70% of the current from Vn0 and 30% from Vn1 in phase, resulting in a synchronized but amplitude-adjusted current signal.

The network connections and parameter implementation syntax for the polynomial weighted sum controlled current sources are as follows:

(2) F_px xx xxx POLY(2) Vn0 Vn1 0 0.7 0.3

F_px denotes a controlled current source, with subsequent nodes indicating its connection points. In the POLY statement, the number inside the parentheses specifies the number of polynomial terms, where the first term is the constant coefficient, and the last two terms represent the proportional factors of the controlling currents. The polynomial weighted sum approach combines control currents from Vn0 and Vn1 with different weighting coefficients, effectively increasing the variation randomness of the synchronous current load and enhancing the diversity and realism of the simulation model. For example, suppose the current on Vn0 is driven by 30 bits switching from 0 to 1, 20 bits switching from 1 to 0, and 14 bits remaining steady at a given transition moment. Meanwhile, the current on Vn1 is driven by 40 bits switching from 0 to 1, 10 bits switching from 1 to 0, and 14 bits remaining steady at the same moment. If these currents are combined using weighting coefficients of 70% for Vn0 and 30% for Vn1 to form F_p02, the equivalent current corresponds to 33 bits switching from 0 to 1, 17 bits switching from 1 to 0, and 14 bits steady. The other polynomial weighted sum CCCS elements are constructed similarly but use different weighting combinations, ensuring that each pseudo interconnect unit’s synchronous current source exhibits pseudo-randomness and maintains distinctiveness. However, one minor drawback of this method is that, after weighted superposition of currents from multiple sources, the resulting total current may correspond physically to a fractional number of bit transitions—for example, if Vn0 consists of 22 bits switching 0→1, 32 bits switching 1→0, and 10 bits steady, and Vn1 consists of 18 bits switching 0→1, 35 bits switching 1→0, and 11 bits steady, then after 70% and 30% weighting, F_p02 effectively corresponds to 20.8 bits switching 0→1, 32.9 bits switching 1→0, and 10.3 bits steady. Although this fractional-bit transition scenario has no direct physical correspondence in real circuits, since the total current is a linear superposition of all switching bit currents, theoretical analysis shows the maximum error is less than 0.5-bit transitions, and this error is pseudo-randomly distributed. Therefore, its impact on the accuracy of PDN noise modeling and PSIJ analysis results can be considered acceptable.

### 3.3. Delayed Polynomial Weighted Sum CCCS Synchronous Current Load and Simulation Configuration Scheme

**Figure 8:**
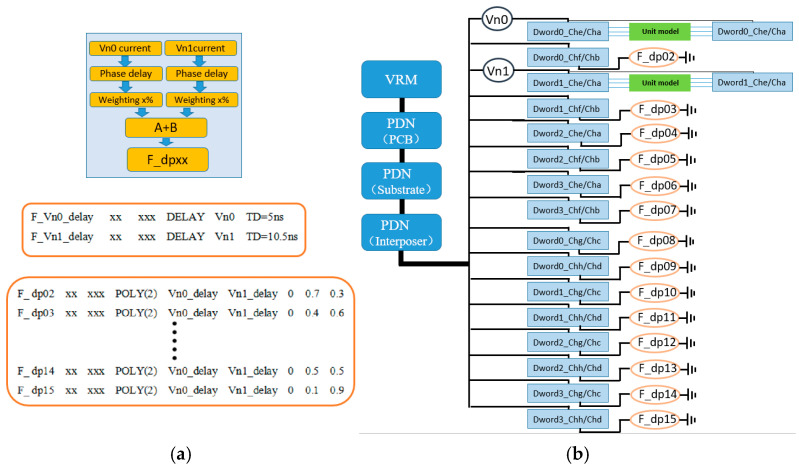
(**a**) Delayed polynomial weighted sum CCCS synchronous current load and (**b**) simulation configuration scheme.

As shown in Figure 8, the simulation scheme for the delayed polynomial weighted sum CCCS synchronous current load still generates the final controlled current load by weighted superposition of multiple control currents. The difference lies in applying a phase delay to the currents on Vn0 and Vn1 before performing the weighted summation. To ensure that the current changes remain synchronized with bit toggling, the delay times for Vn0 and Vn1 currents must be integer multiples of the unit interval (UI). For example, at an HBM2 data rate of 2 Gbps, 1 UI corresponds to 0.5 ns, so a delay of 10 UI equals 5 ns. Additionally, the delay times for Vn0 and Vn1 must differ to reflect the distinction of this method compared to the second method. By delaying the two currents by different amounts before weighted summation, this approach further enhances the randomness of the synchronous current load variation. The implementation syntax for the delayed current sources is as follows:

(3) F_Vn0_delay xx xxx DELAY Vn0 TD=5 ns

(4) F_Vn1_delay xx xxx DELAY Vn1 TD=10 ns

The delayed current sources are combined using the polynomial weighted sum approach described in Method 2 to form the final synchronous current load. Since the specific implementation details are identical to those of Method 2, they will not be repeated here. Both Method 3 and Method 2 aim to enhance the randomness in the synchronous current load variation for simulation purposes. However, Method 3 introduces delays on the control sources, adding an additional dimension of randomness. This improves model flexibility and simulation accuracy, albeit with relatively higher implementation complexity.

## 4. Simulation Comparison and Results Analysis

**Figure 9:**
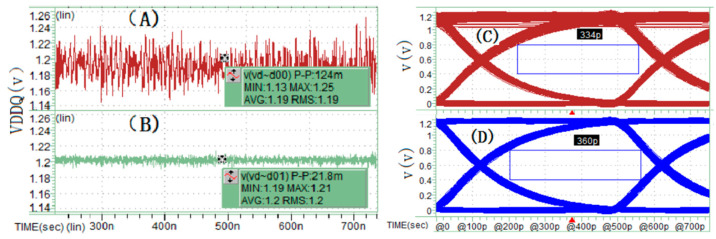
(**A**,**C**) Power noise and DQ15 eye width simulation results for Dword0_Che/Cha unit group using the proportional linear current load scheme. (**B**,**D**) Power noise and DQ15 eye width simulation results for Dword0_Che/Cha unit group with synchronous current loads removed.

This section presents three sets of simulation comparison and analysis results. The first set includes two simulation cases: one employs the proportional CCCS synchronous current load scheme (Figure 6) on the pseudo interconnect units, and the other removes all CCCS synchronous current loads (i.e., no load on the pseudo interconnect units). This comparison aims to verify the impact of current loads generated by HBM Dword signal toggling on power supply SSN and the resulting PSIJ on the signal eye diagram during SSO and SSI simulations. Figure 9 shows the simulation results. Using PRBS11 pseudorandom codes with different seeds, the I/O devices are driven at a transmission rate of 2 Gbps (UI = 500 ps) in transient SPICE simulations. The model incorporates the C-die and R-die parameters of the I/O. Results indicate that adding synchronous current loads on the pseudo interconnect units causes the PDN power noise peak to reach 124 mV, with an eye diagram width of 334 ps. In contrast, removing the 14 synchronous current loads significantly reduces the power noise to 21.8 mV, and the eye diagram width increases to 360 ps. This is attributed to the substantial reduction in dynamic current load on the PDN, which lowers the power noise. The influence of varying SSN levels on PSIJ and consequently on eye width is clearly reflected in the simulation outcomes. This group of simulation results demonstrates that in SSO and SSI simulations of full-channel HBM package interconnects, if only a subset of interconnect units undergo source-synchronous signal transmission, the remaining units must be assigned corresponding loads. Otherwise, the simulation results will deviate from the actual full-channel transmission behavior, potentially causing design verification errors.

**Figure 10:**
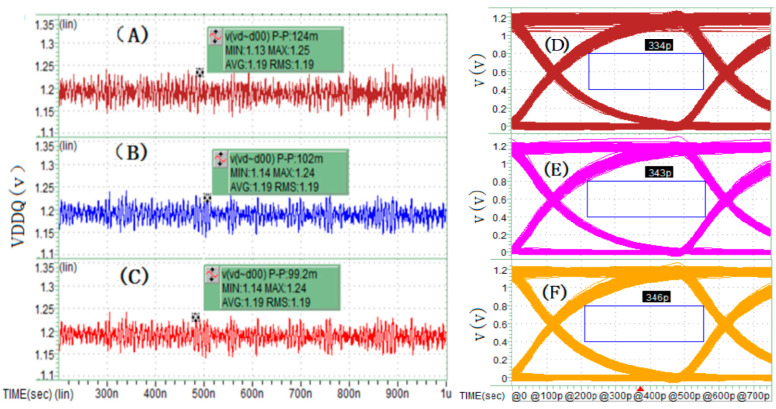
(**A**–**C**) Power noise simulation results of Dword0_Che/Cha unit group for the three synchronous current load methods and (**D**–**F**) DQ15 eye width simulation results of Dword0_Che/Cha unit group for the three synchronous current load methods.

The second set of simulation comparisons aims to demonstrate and illustrate the application effects of the three synchronous current load methods proposed in this paper for pseudo full-channel simulation. This set includes three simulation cases, with configurations corresponding, respectively, to the schemes shown in Figure 6, Figure 7 and Figure 8, while all other simulation conditions remain consistent with those of the first set. The simulation results presented in Figure 10 indicate that the linear synchronous current load method (Figure 6) divides the two real interconnect units and the 14 pseudo interconnect units into two groups, with each group of 8 units sharing identical current loads. This grouping significantly increases the likelihood of peak power noise at the I/O driver terminals, causing greater signal PSIJ. In contrast, the polynomial weighted sum and delayed polynomial weighted sum methods generate synchronous current loads that enhance load randomness, effectively reducing power noise and signal jitter levels. The difference in performance between these latter two methods is minimal in this simulation. Detailed comparative data can be found in Figure 10.

**Figure 11:**
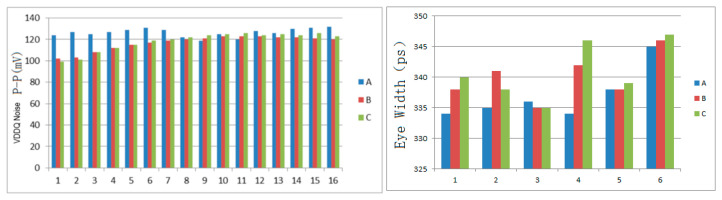
(A) Statistical results of peak-to-peak power noise and eye widths of six DQ for all 16 unit groups using the proportional linear current load scheme, (B) statistical results of peak-to-peak power noise and eye widths of six DQ for all 16 unit groups using the polynomial weighted sum current load scheme, and (C) statistical results of peak-to-peak power noise and eye widths of six DQ for all 16 unit groups using the delayed polynomial weighted sum current load scheme.

For the three synchronous current load generation methods proposed in this paper for pseudo full-channel synchronous signal transmission simulation, it is fundamentally impossible to definitively determine which method will always yield the worst or best simulation results, since these methods inherently involve a degree of randomness, especially the latter two. The generated synchronous current loads depend not only on the original excitation code pattern, simulation duration, number and combination of control sources, and weighting coefficients of the controlled sources, but may also be influenced by factors such as the PDN model and the internal chip CPM model. However, from a probabilistic perspective, different methods of generating synchronous current loads may tend to produce simulation results that are relatively better or worse. For example, when all 16 units use exactly the same current load, the probability of worse results significantly increases. Figure 11 summarizes the peak-to-peak power noise values and the statistical data of eye widths for six randomly selected identical DQ from the second group of simulations using the three synchronous current load methods across 16 interconnect units. The statistics indicate that simulations employing the proportional linear synchronous current load are more likely to exhibit poorer power noise and eye width performance, whereas the other two methods have a lower probability of producing poor results. Nonetheless, this difference is not absolute and only reflects probabilistic variations in the likelihood of better or worse outcomes caused by the different methods.

**Figure 12:**
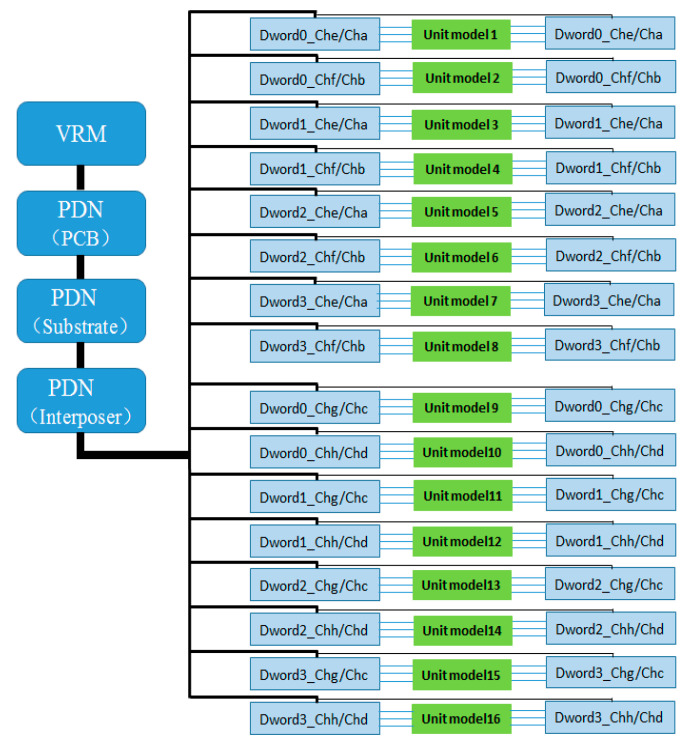
Real full-channel synchronous signal simulation configuration scheme.

The third set of simulation comparisons aims to validate full-channel simulation and the proposed pseudo full-channel simulation. That is very challenging work, because a full 1024-bit SSO or SSI simulation with all interconnect models requires mass computing resources and a very long running time. Furthermore, a large number of coupled interconnect lines greatly increases simulation convergence risk. In fact, modeling complete 1024-bit coupled interconnect lines with EM Solvers is impossible work. So, in the full-channel simulation scheme referenced in Figure 12, 16 interconnect unit models are extracted with the EM Solver separately; each unit model is a s192p S-parameters with 96 traces of two Dword interconnects (48 traces per Dword). For the simulation to be finished successfully in time, each s192p S-parameter (391 MB) model has to be translated to the broadband SPICE model (63 MB).

**Figure 13:**
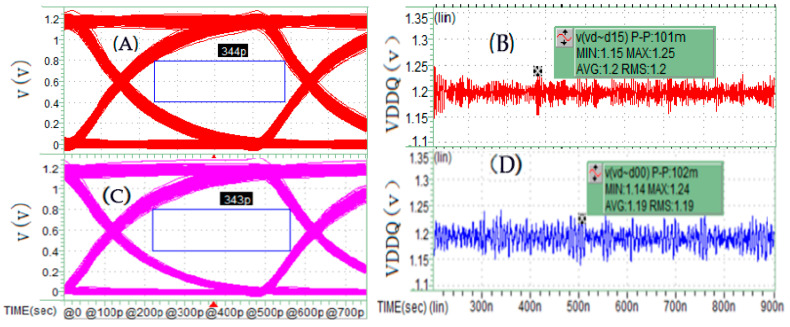
(**A**,**B**) DQ15 eye width and power noise simulation results for Dword0_Che/Cha unit group for real full-channel simulation; (**C**,**D**) DQ15 eye width and power noise simulation results for Dword0_Che/Cha unit group for pseudo full-channel Poly CCCS synchronous current load method.

Figure 13A,B is one of the real full-channel simulation results, as a contrast, the corresponding Figure 13C,D of the Poly CCCS synchronous current load method is presented below. The key performance—eye width and PDN noise of the SSO/SSI simulation are nearly similar. So, we can conclude that the pseudo full-channel simulation with CCCS synchronous current load methods is feasible.

The main value of the pseudo full-channel simulation is efficiency. In the simulation practice, real full channel simulation produces about 10 bits UI (unit interval) results in time domain at 2 Gbps per natural hour utilizing a 2.4 GHz-48-core processor with 512 GB of memory. If we use PRBS11 running 2048 bits, the real full channel simulation will cost about 200 h, which is about 8 days. But the pseudo full-channel simulation with the 2+14 scheme, under the same simulation conditions using PRBS11 running 2048 bits, just costs about 12 h, which is 0.5 days. Compared with real full-channel simulation, the pseudo full-channel simulation runtime reduced by 93%, utilizing a 2.4 GHz, 48-core processor with 512 GB of memory.

## 5. Conclusions

SSO and SSI simulations for HBM synchronous signal transmission are essential techniques for verifying the interconnect design between HBM and AI computing chips. The efficiency, feasibility, accuracy, scalability, and extensibility of these simulation methods have a significant impact on product design and development. The pseudo full-channel signal integrity simulation method for HBM package interconnects proposed in this paper allows for flexible configuration of the number of real interconnect model unit groups and pseudo interconnect units (synchronous current load units), according to factors such as project design cycle, simulation resource constraints, interconnect structure characteristics, and simulation conditions. The 2+14 configuration adopted in this work is merely one possible implementation and can be expanded or modified for different combinations based on specific requirements. In terms of constructing synchronous current loads, this paper introduces three generation methods, each demonstrating certain probabilistic trends in simulation results. These methods can be used individually, in combination, or extended into additional forms. As HBM continues to gain widespread adoption across high-performance computing fields, with increasing parallel interconnect widths (e.g., 2048 bits in HBM4) and growing data transfer rates, challenges such as SSN and PSIJ will become more prominent, imposing stricter demands on simulation accuracy and efficiency. The greater significance of this work lies in presenting a simulation methodology that is both practical and scalable, aiming to contribute to the advancement of HBM interconnect design and signal integrity simulation techniques, while also offering a reference framework and practical approach for engineers facing similar simulation requirements.

## Data Availability

Data are contained within the article.
